# Characterization of the biological and transcriptomic landscapes of bone marrow-derived mesenchymal stem cells in patients with multiple myeloma

**DOI:** 10.1186/s12935-024-03308-2

**Published:** 2024-03-27

**Authors:** Yu Lu, Chaohui Zheng, Wenxia Zhang, Xuan Liu, Ziwei Zhou, Zhenzhen Wang, Huan Hua, Zhengrong Song, Xuejun Zhang, Shuyi Liu, Leisheng Zhang, Fuxu Wang

**Affiliations:** 1 Department of Hematology, Hebei Key Laboratory of Hematology, The Second Hospital of Hebei Medical University, Shijiazhuang, 050000 China; 2 Department of Otolaryngology, The Second Affiliated Hospital of Fujian Medical University, Quanzhou, 362000 China; 3 Science and Technology Innovation Center, The Fourth People’s Hospital of Jinan & The Teaching Hospital of Shandong First Medical University, 50 Shifan Road, Tianqiao District, Jinan, 250031 China; 4 National Health Commission (NHC) Key Laboratory of Diagnosis and Therapy of Gastrointestinal Tumor, Gansu Provincial Hospital, Lanzhou, 730000 China

**Keywords:** Multiple myeloma (MM), BM-MSCs, Biological signatures, Transcriptomic variation, Cellular viability

## Abstract

**Background:**

Mesenchymal stem/stromal cells (MSCs) have been acknowledged as the most important stromal cells in the bone marrow (BM) microenvironment for physiologic hematopoiesis and the concomitant hematologic malignancies. However, the systematic and detailed dissection of the biological and transcriptomic signatures of BM-MSCs in multiple myeloma (MM) are largely unknown.

**Methods:**

In this study, we isolated and identified BM-MSCs from 10 primary MM patients and 10 healthy donors (HD). On the one hand, we compared the multifaceted biological characteristics of the indicated two BM-MSCs, including biomarker expression pattern, multilineage differentiation potential, stemness and karyotyping, together with the cellular vitality and immunosuppressive property. On the other hand, we took advantage of RNA-SEQ and bioinformatics analysis to verify the similarities and differences at the transcriptomic level between MM-MSCs and HD-MSCs.

**Results:**

As to biological phenotypes and biofunctions, MM-MSCs revealed conservation in immunophenotype, stemness and differentiation towards adipocytes and chondrocytes with HD-MSCs, whereas with impaired osteogenic differentiation potential, cellular vitality and immunosuppressive property. As to transcriptomic properties, MM-MSCs revealed multidimensional alterations in gene expression profiling and genetic variations.

**Conclusions:**

Overall, our date systematic and detailed reflected the multifaceted similarities and variations between MM-MSCs and HD-MSCs both at the cellular and molecular levels, and in particular, the alterations of immunomodulation and cellular viability of MM-MSCs, which wound benefit the further exploration of the pathogenesis and new drug application (NDA) of multiple myeloma from the view of BM-MSCs.

**Supplementary Information:**

The online version contains supplementary material available at 10.1186/s12935-024-03308-2.

## Background

Multiple myeloma (MM), a hematologic malignancy with multi-organ threatening complications, has been characterized by the spectrum of plasma cell dyscrasias and monoclonal gammopathy, together with abnormalities in the bone marrow microenvironment (e.g., stromal cells, osteoclastogenesis) [1]. Despite the considerable progress in pathogenesis and targeted therapies, the outcomes of patients with MM are still far from satisfaction after receiving conventional treatment, hematopoietic stem cell transplantation (HSCT) and immunotherapy due to the deficiency of systematic and detailed investigation of the cellular and molecular landscapes [2–4].

Mesenchymal stem/stromal cells (MSCs) are key component in the microenvironment with unique immunosuppressive and hematopoietic-supporting properties as well as multilineage differentiation potential towards adipocytes, osteoblasts, and chondrocytes [5, 6]. During the past decades, we and other investigators in the field have devoted to fulfilling the feasibility of MSC-based cytotherapy for a variety of refractory and recurrent diseases, including Crohn’s disease-related enterocutaneous fistula [7], aplastic anemia [8], premature ovarian failure (POF) [9], acute-on-chronic liver failure (ACLF) [10], Alzheimer's disease [11], osteoarthritis [12] and chronic obstructive pulmonary disease (COPD) [13]. Simultaneously, MSCs have caught increasing attention in the field of hematologic malignancies, especially for their immunodysfunction and tumorigenicity in acute leukemia [14–16]. In recent years, the multifaceted alterations and deficiency of MSCs in a variety of diseases have also been unremittingly verified such as acute myelogenous leukemia[17], acquired aplastic anemia [18], myelodysplastic syndrome (MDS) [19], type 2 diabetes mellitus[20], and immune thrombocytopenia (ITP) [21]. Taken together, considerable literatures have highlighted the pivotal role of MSCs in disease management and the concomitant pathogenesis via trans- or direct-differentiation, secretion (e.g., cytokines, exosomes, microvesicles), dual immunomodulation, and orchestrating the constitutive microenvironment [22, 23]. However, the systematic and detailed information of the intrinsic characteristics of BM-MSCs in multiple myeloma is still largely unknown.

For the purpose, we identified BM-MSCs from MM patients (MM-MSCs) and healthy donors (HD-MSCs), and conducted multidimensional comparison of the biological signatures and transcriptomic properties. On the one hand, MM-MSCs revealed deficiency in osteogenic differentiation potential and cellular vitality, decline in suppressing CD4^+^ T lymphocytes and increase in promoting Th17 cells compared with HD-MSCs, whereas no differences in immunophenotypes, adipogenic and chondrogenic differentiation, pluripotency-related biomarker expression, chromosome karyotype. On the other hand, our data intuitively reflected the multifaceted similarities and differences in gene expression profiling and the spectrum of genetic variations. Taken together, our findings indicated the conservation and alterations between MM-MSCs and HD-MSCs both at the cellular and molecular levels, which would collectively benefit the further investigation of the pathogenies and therapeutic strategies in future.

## Methods

### Patients

Bone marrow samples were obtained from 10 patients with primary treated MM (male = 3; female = 7; age: 35–59 year-old, 43.3 ± 6.961 year-old) and 10 HDs (male = 7; female = 3; age: 34–56 year-old, 49.9 ± 7.549 year-old). All participants signed informed consent, and all procedures were approved by the Ethical Committee of The Second Hospital of Hebei Medical University according to the guideline of the Declaration of Helsinki (Approval Number: 2022-R302). The detailed information of MM patients (MM) and healthy donors (HDs) was available in Additional file 4: Table S5.

### Cell culture and passage

MM-MSCs and HD-MSCs were isolated from bone marrow-derived mononuclear cells (BM-MNCs) by utilizing the Ficoll-based (DongFangHuaHui, China) density gradient centrifugation at room temperature (RT) as we described before [18, 24]. The indicated MM-MSCs and HD-MSCs at the same passage were cultured in MSCs serum-free medium (Jingmeng StemCell, China). The indicated MM-MSCs and HD-MSCs were cultured at 37 ℃, and 5% CO_2_, and the medium was replaced every 3 days. The indicated BM-MSCs at the same passage (ranging from passage 3 to passage 8) were washed twice with DPBS (Sigma-Aldrich, USA) and detached with 0.25% Trypsin/EDTA (Gibco, USA) for passage when reached 80–90% confluence, and then collected by centrifugation at 300 × g. The harvested cells were mixed using 0.4% Typan blue (Sigma-Aldrich, USA) for cell counting under an inverted microscope (ZEISS, Germany).

### Flow cytometry (FCM) assay

FCM assay was performed as we reported before with several modifications [8, 12]. In details, MSCs cells were washed twice with DPBS (Sigma-Aldrich, USA) and labeled with fluorescence-conjunct antibodies (e.g., CD3, CD4, CD8, CD44, CD45, CD73, CD90, CD105, CD34, HLA-DR) in dark for 30 min. After that, the MSCs were washed with DPBS and resuspended in 0.2% BSA (Sigma-Aldrich, USA). Finally, the cells were turned to FACS Canto II (BD Biosciences) and FlowJo 10.0 (Tree Star, USA) for analysis. The list of the antibodies was available in Additional file 1: Table S1.

### Quantitative real-time polymerase chain reaction (qRT-PCR) assay

The qRT-PCR assay was conducted as we recently described with several modifications [8, 18]. In details, MM-MSCs and HD-MSCs were washed twice with DPBS, and mRNAs were extracted by using E.Z.N.A. Total RNA kit II (Omega, China) according to the manufacturer’s instruction. The mRNAs were quantified and synthesized into cDNA with SureScript™ First-strand cDNA Synthesis kit (GeneCopoeia, China). Then, qRT-PCR was performed by utilizing rotor gene Q and All-in-One™ qPCR Mix (GeneCopeia). The primer sequences were available in Additional file 1: Table S2.

### CCK-8-bassed cell proliferation analysis

CCK-8 assay was performed for cell proliferation assessment as we recently described with several modifications [8, 25]. Briefly, HD-MSCs and MM-MSCs were seeded in 96-well plates at a density of 2.5 × 10^3^/well, and OD450 values were detected by using the cell counting kit-8 (CCK-8) (BOSTER, China) and the microplate reader (Bio-Rad, USA) under absorbance at 450 nm at the indicated time points (0 h, 24 h, 72 h, 120 h) according to the manufacturer’s instruction.

### Karyotype analysis

Chromosome karyotyping was conducted as we described before [8, 18]. Briefly, MM-MSCs and HD-MSCs in meta-phase were treated with colchicine and made into chromosome suspensions, and the G-bands were developed using the G-banding technique (ZEISS). The morphogen of the indicated BM-MSCs were recorded under the Olympus DP71 microscope (Tokyo, Japan).

### Multi-lineage differentiation of MSCs

To compare the multi-lineage differentiation capacity of MM-MSCs and HD-MSCs, 5 × 10^4^ cells were seeded in 12-well plates in MSCs culture medium for 3 days. When cells reached 80% confluence, the medium was changed into adipogenic, osteogenic, and chondrogenic differentiation medium (Stem Cell Technologies, USA), respectively. 3 weeks later, the BM-MSC-derived adipocytes, osteoblasts, and chondrocytes were identified by Oil red O staining, Alizarin Red S staining, and Alcian Blue staining, respectively. Meanwhile, the aforementioned BM-MSCs-derived cells were lysed by TRIZol reagent (Sigma-Aldrich, USA), and then turned to qRT-PCR assay for quantification. The primer sequences of the indicated genes were available in Additional file 1: Table S2.

### Apoptotic detection of MSCs

For apoptotic detection, MM-MSCs and HD-MSCs were turned to the Annexin V Apoptosis Detection Kit (Sigma-Aldrich, USA) as we previously reported [18, 25]. According to the manufacturer’s instructions, a total number of 1 × 10^6^ cells were washed with 1 × PBS (Gibco, USA) and resuspended in 100 µL 1 × Binding Buffer. After that, the MSCs were incubated in PIand Annexin V-FITC solution at 4 ℃ for 20 min in dark. The proportions of apoptotic cells in the aforementioned MSCs were detected with FACS Canto II (BD, USA) and FlowJo 10.0 (Tree Star, USA).

### Cell cycle assessment

The quantification of cell cycle was accomplished based on DNA content assay as we described before [8, 25]. In details, 2 × 10^5^ MM-MSCs or HD-MSCs were harvested and fixed by 70% pre-cooled ethanol at 4 ℃ for overnight. Subsequently, the MSCs were washed by DPBS and incubated with RNaseA at 37 ℃ for 30 min. Finally, MSCs were labeled with Propidium iodide (PI) staining solution and detected by FACS Canto II (BD, USA) and FlowJo 10.0 (Tree Star, USA).

### Mixed lymphocyte co-culture (MLC)

To compare the immunomodulatory property of MM-MSCs and HD-MSCs, we conducted MLC assay as described before [5, 18]. For preparation of peripheral blood-derived mononuclear cells (PBMCs), we utilized the Ficoll-based (DongFangHuaHui, China) density gradient centrifugation. Then, the CD3 microbeads (Miltenyi Biotec)-based magnetic activated cell sorting (MACS) was used for CD3^+^ T cell enrichment from PBMCs. As to MLC assay, 2 × 10^4^ MM-MSCs or HD-MSCs were mixed with 1 × 10^5^ CD3^+^ T cells in a 96-well plate in 1640 basal medium (Gibco) supplemented with 10% FBS (OriCell, China). Meanwhile, CD3/CD28 Dynabeads (the Dynabeads^™^ CD3/CD28 In Vitro T Cell Expansion Magnetic Bead Reagent, ThermoFisher, USA) were introduced to stimulate CD3^+^ T cell activation. After 3 days, the number of CD3^+^ T cells was counted, and the percentages of the subpopulations were verified by flow cytometry (BD FASC Canto II) after labeling with the indicated antibodies (CD3, CD4, CD183, CD196). The list of the antibodies was available in Additional file 1: Table S1.

### RNA-seq and bioinformatic analysis

Total mRNAs of HD-MSCs (passage 3, n = 3 independent samples) and MM-MSCs (passage 3, n = 3 independent samples) were extracted by using the TRIZol reagent (Sigma-Aldrich, USA) according to the manufacturer’s instruction, and quantified by NanoDrop (ThermoFisher, USA). After that, the mRNAs were turned to Novogene (Tianjin, China) for RNA-sequencing (RNA-seq), and the multifaceted bioinformatics analyses (e.g., GOBP, KEGG, HeatMap, PCA, GSEA) were accomplished by using the indicated databases and online platforms as we recently described [12, 17, 24]. The gene expression profiling was available in Additional file 2: Table S3, and the genetic variations were available in Additional file 3: Table S4.

### Statistical analysis

All statistical analyses were performed with the Prism 8.0 (GraphPad Software) as we reported before [18, 24, 25]. In details, unpaired t -test was used for comparison between the two groups, and one-way ANOVA was used for comparing the multiple unpaired groups. Statistically significant differences were considered only when the P-value was less than 0.05. All data were shown as mean ± SEM (N = 3 independent experiments).

## Results

### MM-MSCs revealed no differences in immunophenotype and stemness with HD-MSCs whereas with impaired osteogenic differentiation potential

**Fig. 1:**
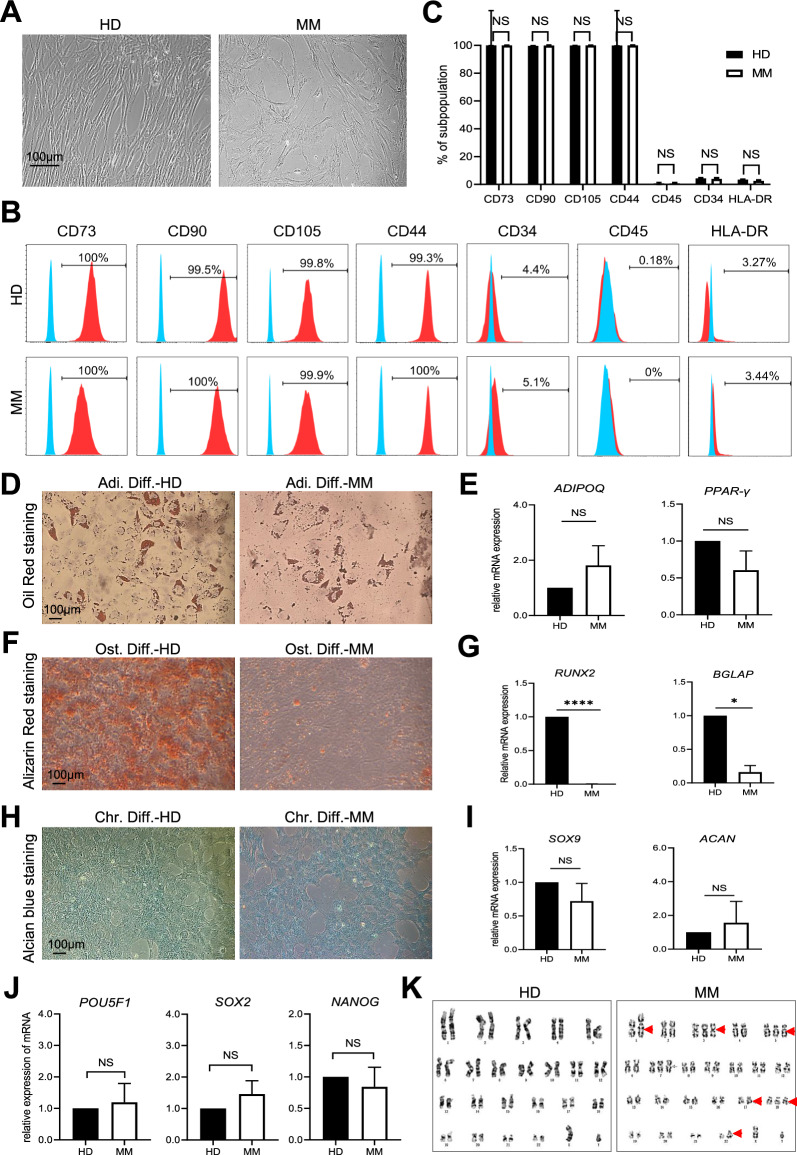
MM-MSCs showed similarities in immunophenotype with HD-MSCs but variations in osteogenic differentiation. **A** Representative phase contrast images of BM-MSCs derived from healthy donors (HD-MSCs) and MM patients (MM-MSCs), respectively. Scale bar = 100 μm. **B**, **C** Representative flow cytometry (FCM) diagrams **B** and statistical analysis **C** of biomarkers in HD-MSCs and MM-MSCs. **D**, **E** Adipogenic differentiation assessment of HD-MSCs and MM-MSCs by Oil Red O staining **D** and qRT-PCR analyses of adipogenic differentiation-related genes (*ADIPOQ*, *PPAR-γ*) (**E)**. **F**, **G** Osteogenic differentiation of HD-MSCs and MM-MSCs by Alizarin Red S staining **F** and qRT-PCR analyses of osteogenic differentiation-related genes (*RUNX2, BGLAP*) (**G)**. **H**, **I** Chondrogenic differentiation of HD-MSCs and MM-MSCs by Alician Blue staining **H** and qRT-PCR analyses of chondrogenic differentiation-related genes (*SOX9*, *ACAN*) (**I)**. **J** qRT-PCR analyses of stemness-related (*POU5F1*, *SOX2*, *NANOG*) genes in HD-MSCs and MM-MSCs. **K** Karyotypic analysis of HD-MSCs and MM-MSCs. All data were shown as mean ± SEM (N = 3 independent experiments). *NS* not significant, *P < 0.05; ****P < 0.0001

To illuminate the systematic and detailed information of the similarities and differences between MM-MSCs and HD-MSCs, we isolated and identified BM-MSCs from 12 patients with primary-treated multiple myeloma (MMs) and the concomitant 9 healthy donors (HDs). Morphologically, both MM-MSCs and HD-MSCs revealed typical spindle-like shape (Fig. 1A). Similarly, with the aid of FCM assay, no differences were observed between MM-MSCs and HD-MSCs in biomarker expression pattern (P > 0.05) (Fig. 1B, C).

With the aid of multi-lineage differentiation assay, we found MM-MSCs revealed no differences with HD-MSCs in adipogenic and chondrogenic differentiation whereas with sharp decline in osteogenic differentiation potential instead (Fig. 1D–I). Meanwhile, to evaluate the stemness of the aforementioned BM-MSCs, we conducted qRT-PCR assay of pluripotency-associated biomarkers (*POU5F1*, *SOX2*, *NANOG*) and observed no differences between them (Fig. 1J). Additionally, as shown by G-banding analysis, MM-MSCs exhibited a variety of chromosomal abnormalities compared with HD-MSCs (Fig. 1K). Collectively, we noticed the conservation in immunophenotypes and stemness between MM-MSCs and HD-MSCs, together with the variations in multi-lineage differentiation and karyotyping.

### MM-MSCs manifested multidimensional variations with HD-MSCs in gene expression profiling

**Fig. 2:**
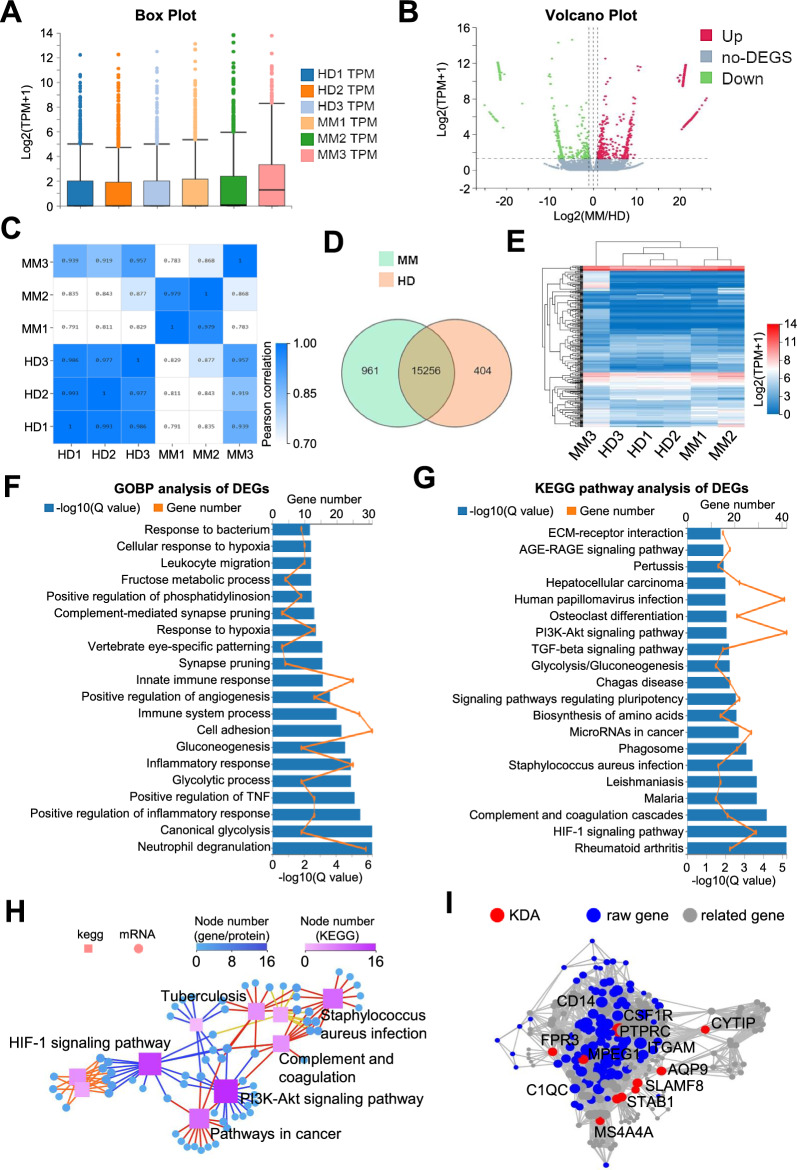
Gene expression pattern of HD-MSCs and MM-MSCs. **A**, **B** The Box Plot **A** and Volcano Plot **B** of gene expression profiling in HD-MSCs (HD1, HD2, HD3) and MM-MSCs (MM1, MM2, MM3) based on log_2_ (TPMM + 1), respectively. **C** Correlation analysis of HD-MSCs and MM-MSCs. **D** The Venn Map analysis of the genes expressed in HD-MSCs and MM-MSCs. **E** The HeatMap diagram of gene expression profiling in HD-MSCs and MM-MSCs. **F**, **G** Gene ontology biological process (GOBP) analysis **F** and KEGG pathway analysis **G** of differentially expressed genes (DEGs) between HD-MSCs and MM-MSCs. **H** KEGG correlation **H** and KDA **I** analyses of the DEGs between HD-MSCs and MM-MSCs

Having verified the biological characteristics at the cellular level, we next turned to assess the properties of HD-MSCs and MM-MSCs at the molecular level. By conducting RNA-SEQ analysis, we intuitively observed the variations in gene expression pattern according to the bar chart of box plot and volcano plot (Fig. 2A, B). Based on Pearson correlation assay, we found MM-MSCs from different individuals (MM1, MM2, MM3) revealed greater variations over those of the HD-MSCs (HD1, HD2, HD3) (Fig. 2C). In details, as shown by Venn Map, a total number of 1356 differentially expressed genes (DEGs) (961 upregulated DEGs, 404 downregulated DEGs) and 15256 non-DEGs were observed between MM-MSCs and HD-MSCs, which were further confirmed by HeatMap diagram (Fig. 2D, E).

Subsequently, we tried to investigate the underlying biological significances of the DEGs, we took advantage of the gene ontology biological process (GOBP) and KEGG pathway assay. On the one hand, we noticed the enrichment of metabolism- and immunomodulation-associated GOBP, including canonical glycolysis, innate immune response, positive regulation of TNF and inflammatory response (Fig. 2F). On the other hand, the DEGs were involved in a variety of signaling pathways such as HIF-1 signaling pathway, PI3K-Akt signaling pathway, and TGF-β signaling pathway, which were further confirmed by the pathway interaction assay (Fig. 2G, H). Additionally, according to the KDA assay, we could further observe the relative spatial relationship of the individual DEGs and the correlations among them (Fig. 2I). Taken together, MM-MSCs exhibited multidimensional variations in gene expression profiling compared with HD-MSCs.

### MM-MSCs showed variations in the spectrum of biofunction and genetic mutation pattern

**Fig. 3:**
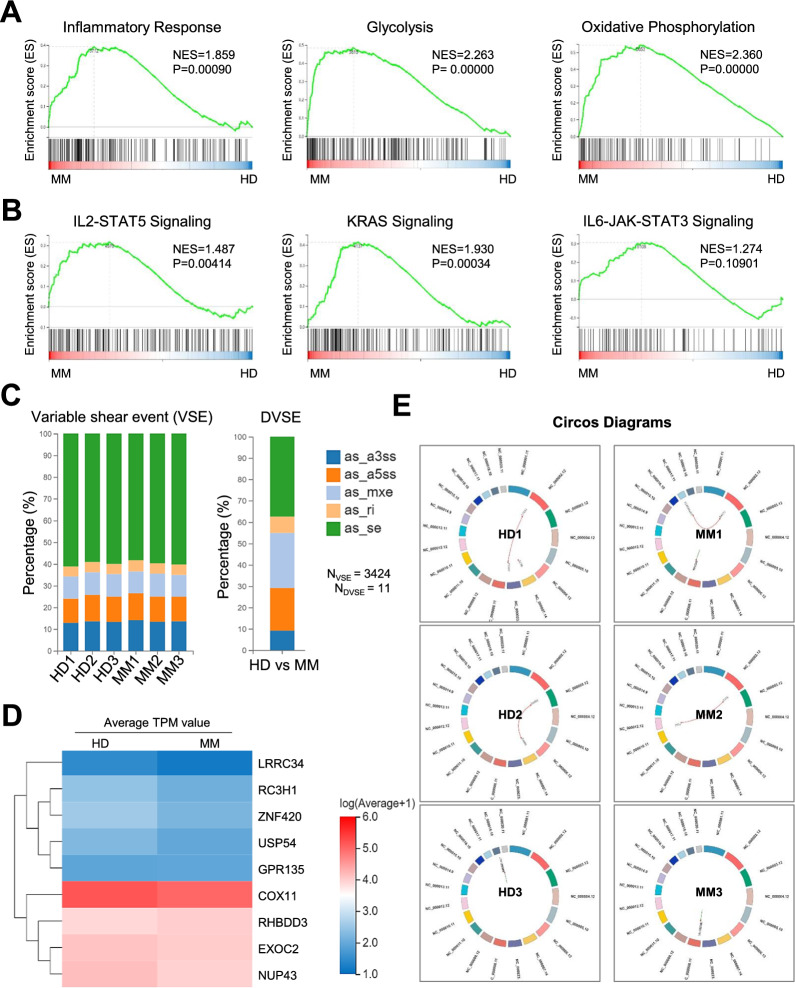
The biofunction of gene sets and genetic variation spectrum between AML-MSCs and HD-MSCs. **A**, **B** Gene set enrichment analysis (GSEA) of the differentially functional gene sets including the bioprocesses **A** and signaling pathways **B** between HD-MSCs and MM-MSC, respectively. **C** The variations in genes with variable shear event and DVSE between HD-MSCs and MM-MSCs. **D** The HeatMap diagram of the indicated genes with DVSE between HD-MSCs and MM-MSCs. **E** Circos diagrams showed the variations of loci regional distribution and gene fusion events between HD-MSCs and MM-MSCs

To further estimate the biofunction and genetic variations between HD-MSCs and MM-MSCs, we turned to gene set enrichment analysis (GSEA) and noticed that the specific enrichment of the genes was mainly involved in inflammatory response, glycolysis and oxidative phosphorylation, which was consistent with the GOBP assay (Fig. 3A). Similarly, the genes were mainly involved in IL2-STAT5 signaling (P = 0.00414) and KRAS signaling (P = 0.00034) rather than IL6-JAK-STAT3 Signaling (P = 0.10901) (Fig. 3B).

In the meantime, we attempted to illuminate the potential similarities and differences in genetic variations between the aforementioned MSCs. Generally, MM-MSCs and HD-MSCs showed similarities in the proportions of the indicated variable shear events (VSEs), including the alternative 3′ splicing site (as_a3ss), alternative 5′ splicing site (as_a5ss), mutually exclusive exon (as_mxe), retained intron (as_ri), and skipped exon (as_se) subtypes (Fig. 3C). Of the 3424 genes with VSE, only 11 ones were differentially VSEs (DVSEs) between MM-MSCs and HD-MSCs (Fig. 3C). Furthermore, 9 of the indicated 11 genes showed variations in expression at transcriptional level according to the HeatMap analysis (Fig. 3D). Additionally, as shown by the Circos diagrams, the loci distribution and expression pattern of the indicated genes with VSE in the chromosome between MM-MSCs and HD-MSCs was intuitively presented (Fig. 3E). Collectively, these data indicated the gene expression and genetic variation pattern together with the potential influences to the functional deficiency of MM-MSCs.

### MM-MSCs showed increase in apoptosis but decline in proliferation and cell cycle

**Fig. 4:**
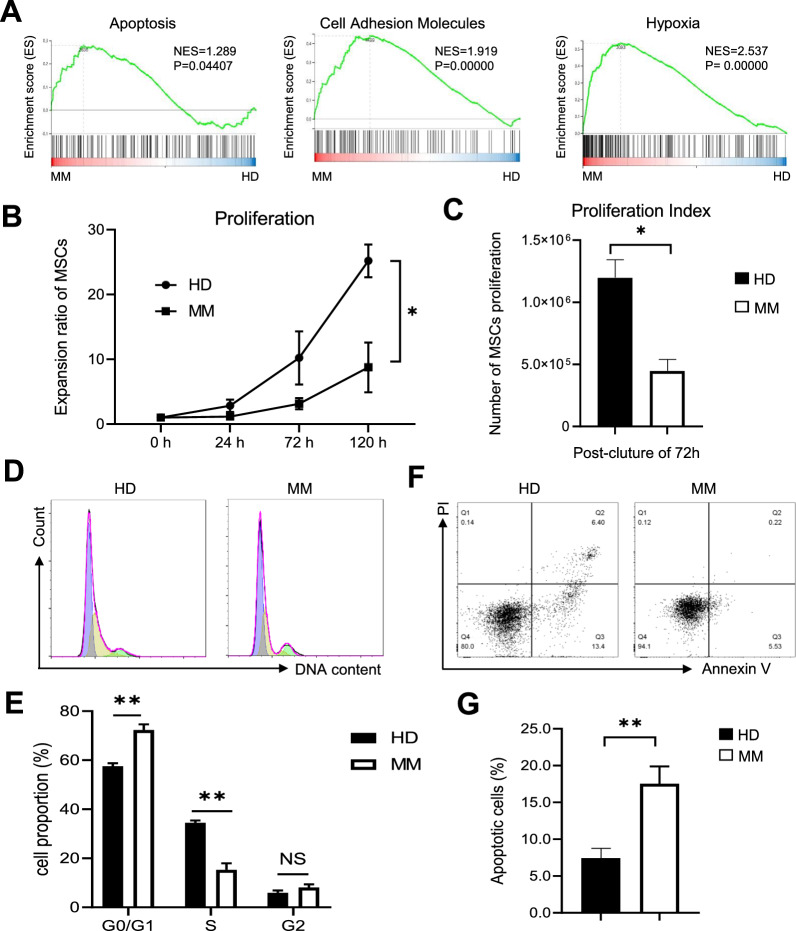
The comparison of the cellular vitality between HD-MSCs and MM-MSCs. **A** GSEA of the differentially functional gene sets including apoptosis, cell adhesion molecules and hypoxia between HD-MSCs and MM-MSCs, respectively. **B** Proliferation curve of HD-MSCs and MM-MSCs at the indicated timepoints according to CCK-8 assay. **C** Statistical analysis of the proliferation index between HD-MSCs and MM-MSCs. **D**, **E** Representative diagrams **D** and statistical analysis **E** of the cell cycle substages (G0/G1, S, G2) between HD-MSCs and MM-MSCs. **F**, **G** Representative diagrams **F** and statistical analysis **G** of the apoptotic subpopulations between HD-MSCs and MM-MSCs. All data were shown as mean ± SEM (N = 3 independent experiments). NS, not significant; *P < 0.05; **P < 0.01

Cellular vitality and homing ability are the prerequisites of MSC-based therapeutics for refractory and recurrent disease administration as well as regenerative medicine [8, 26]. Aiming to dissect the variations of MM-MSCs in cellular vitality, we conducted GSEA and noticed the differences in the enrichment of apoptosis-, cell adhesion molecules- and hypoxia-associated gene sets between MM-MSCs and HD-MSCs (P < 0.05) (Fig. 4A). As shown by the CCK-8-based cumulative growth curve, MM-MSCs displayed sharp decline in cell proliferation compared to HD-MSCs, which was confirmed by the statistical analysis of proliferation index (Fig. 4B, C). Meanwhile, we also observed the alterations of MM-MSCs in cell cycle including the decreased proportion of the subpopulation at the S stage and the contrary tendency in the G0/G1 subset (Fig. 4D, E). In consist with the GSEA prediction, the percentage of apoptotic cells in MM-MSCs was over twofold higher than that in HD-MSCs (P < 0.01) according to the Annexin V and PI staining (Fig. 4F, G). Collectively, MM-MSCs displayed multidimensional variations in cellular vitality when compared to HD-MSCs.

### MM-MSCs revealed decline in suppressing CD3^+^ T cell proliferation whereas enhanced pro-differentiation towards the Th17 subset

**Fig. 5:**
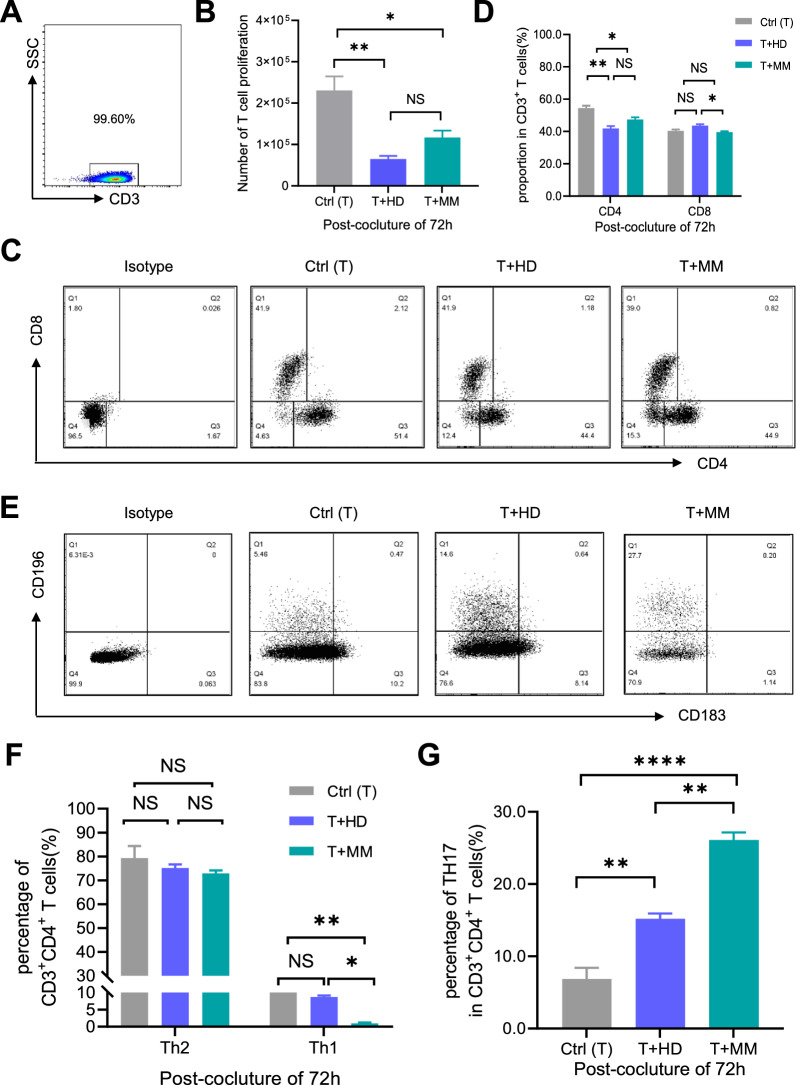
The comparison of the immunosuppressive capacity upon T lymphocytes between HD-MSCs and MM-MSCs. **A** Representative FCM diagram of enriched total CD3^+^ T cells from PBMCs by MACS. **B** T cell counting after 3-day’s culture alone (Ctr) or coculture with HD-MSCs (T + HD) or MM-MSCs (T + MM). **C**, **D** Representative FCM diagram **C** and statistical analysis **D** of CD3^+^CD4^+^ T cells and CD3^+^CD8^+^cells after 3-day’s culture. **E**, **G** Representative FCM diagram **E** and statistical analysis **F** of Th1 (CD3^+^CD4^+^CD183^+^CD196^−^) and Th2 (CD3^+^CD4^+^CD183^−^CD196^−^), together with Th17 (CD3^+^CD4^+^CD183^−^CD196^+^) proportions **G** after 3-day’s culture. All data were shown as mean ± SEM (N = 3 independent experiments). *NS* not significant; *P < 0.05; **P < 0.01; ****P < 0.0001

To further examine the potential alterations of MM-MSCs in immunomodulation, we cocultured the PBMC-derived total CD3^+^ T cells with the indicated MM-MSCs or HD-MSCs (Fig. 5A). According to the statistical analysis, the number of total CD3^+^ T cells and CD3^+^CD4^+^ T cells after 3-day’s coculture with MSCs (T + MM, T + MM) was less than that in the control group (Ctr, culture alone), and the inhibitory effect of MM-MSCs was partially impaired when compared with HD-MSCs (Fig. 5B, D). Interestingly, as shown by the FCM diagrams and statistical analysis, there were no differences in the proportion of CD3^+^CD8^+^ T cells between the Ctr group and the T + HD group (P = 0.0761), whereas with the minor decline in the T + MM group (P = 0.0346) (Fig. 5C, D). Subsequently, the inhibitory effect of MM-MSCs upon T cell differentiation towards Th1 (CD3^+^CD4^+^CD183^+^CD196^−^) rather than Th2 (CD3^+^CD4^+^CD183^−^CD196^−^) was enhanced when compared to HD-MSCs (Fig. 5E, F). Similarly, the Th17 (CD3^+^CD4^+^CD183^−^CD196^+^) differentiation-promoting effect of MM-MSCs was abnormally enhanced when compared to HD-MSCs (Fig. 5E, G). Overall, MM-MSCs showed decline in inhibiting total T cell proliferation and differentiation towards Th1 cells, whereas manifested enhancement in benefiting Th17 activation instead.

## Discussion

As a malignant hematological disease occurs in the elderly [27], MM is characterized by abnormal proliferation of bone marrow plasma cells, together with monoclonal immunoglobulin increase (M-band), multi-organ damage, bone destruction, and impaired renal function [3, 28]. Despite the improvement in median survival time of MM patients attributes to the considerable progresses in treatment strategies (e.g., proteasome inhibitors, immunomodulators) and autologous hematopoietic stem cell transplantation (ASCT), yet the persistent prognosis remains inadequate largely due to the deficiency of systematic and detailed dissection of the pathogenesis [29, 30]. In this study, with the aid of multifaceted biological and transcriptomic analysis, we verified the similarities and variations between MM-MSCs and HD-MSCs at the cellular and molecular levels, which collectively highlighted the involvement and potential influence of BM-MSCs for MM pathogenesis.

MM, a life threatening malignancy of plasma cells, has been demonstrated with excessive osteoclast-mediated bone destruction [31]. During the past decades, we and other investigators have dedicated to disclosing the underlying pathogenesis and therapeutic remedies. For instance, a variety of pivotal factors have been involved during osteoclastic bone resorption, including Dickkopf 1 overexpression, macrophage inflammatory protein-1alpha (MIP-1α) and nuclear factor-kappaB ligand (RANKL) activation [31]. Meanwhile, Wang et al. recently reported the ameliorative effect of apoptotic extracellular vesicles upon MM by restoring Fas-mediated apoptosis [32]. Consistently, our data indicated the unusual increase in the proportion of apoptotic subpopulation and the enrichment of apoptosis-associated gene set in MM-MSCs compared to HD-MSCs, which for the first time systematically highlighted the potential pathogenesis of the incurable MM due to BM-MSC alteration. Notably, our data indicated that MM-MSCs upregulated production of Th17 T cells, which put forward the potential correlations between Th17 cells and myeloma-induced bone disease and immunosuppression. Additionally, we also observed the multifaceted alterations in the cellular viability of MM-MSCs when compared to HD-MSCs. Thus, it’s interesting to further explore the potential correlations and clinical relevance between the molecular pathways and the induction of Th17 cells in future.

For a long period, investigators in the field have committed to explore the potential application of MSCs with different origins for a series of intractable disease management, including umbilical cord-derived MSCs (UC-MSCs) [5], placental tissue-derived MSCs (P-MSCs) [8], adipose tissue-derived MSCs (ASCs) [20], dental pulp-derived MSCs (DPSCs) [33], BM-MSCs [34], supernumerary teeth-derived apical papillary stem cells (SCAP-Ss) [33], embryonic stem cell-derived MSCs (ESC-MSCs) [35, 36] and induced pluripotent stem cell-derived MSCs (iPSC-MSCs) [12]. For example, Rethnam and the colleagues reported the pro- and tumor-suppressive effects of MSCs via reducing the level of MM-derived matrix metalloproteinase-9 (MMP-9) [37].

Notably, due to the pivotal role in the hematopoietic microenvironment and dual immunomodulatory capacity, more and more literatures have emerged to elucidate the potential pathogenesis of MSCs in a variety of disorders such as aplastic anemia [18], acute myelogenous leukemia [17], and type 2 diabetes mellitus [20]. For instance, Spelat et al. and Lemaitre L et al. respectively suggested the involvement of the cytokines (e.g., IL-6, IL-10) in secretome and transcriptome for MM development via facilitating MM cell proliferation [38, 39]. As to MM, talented pioneers in the field have also indicated the diverse abnormalities in the cellular phenotype and distinct genomic profile to a certain extent [40–44]. However, to our knowledge, the systematic and detailed characterization of the biological and transcriptomic properties of BM-MSCs during multiple myeloma is largely unavailable. Herein, by conducting multifaceted analyses, we verified the similarities and variations between MM-MSCs and HD-MSCs from the view of cellular and molecular levels. Moreover, our data prompted the involvement of the potential pathogenic role of MM-MSCs with alterations in osteogenesis and bone destruction, metabolism (e.g., glycolysis, fructose metabolic process, gluconeogenesis) and immunoregulatory response (e.g., positive regulation of TNF, inflammatory response, Th17 cell activation), together with hyperactivation of signaling cascades (e.g., IL2-STAT5 signaling, KRAS signaling). These data were consistent with the previous report that MSCs functioned an important role in both the leukemic process of multiple myeloma and physiological hematopoiesis [5, 45]. Additionally, it would be of great interesting to compare the omics signatures between primary HD-MSCs and MM-MSCs by utilizing the single-cell RNA-sequencing (scRNA-seq) technology in further investigators.

Collectively, our findings illuminated the multidimensional similarities and variations between MM-MSCs and HD-MSCs, which supplied new references to further explore the underlying molecular mechanism of multiple myeloma and would benefit the development of MSC-based cytotherapy for facilitating hematopoietic stem cell transplantation and hematopoietic reconstitution in future.

## Conclusion

Overall, with the aid of biological and transcriptomic analysis, we systematically and detailed dissected the similarities and differences between MM-MSCs and HD-MSCs. These findings will provide new clues and references for the further exploration of MSC-based pathogenesis and remedies for patients with multiple myeloma.

### Supplementary Information

**Additional file 1: Table S1.** The list of the antibodies. **Table S2.** The primer sequences for q RP-PCR assay.

**Additional file 2: Table S3.** Gene Expression Profiling between HD and MM.

**Additional file 3: Table S4.** Genetic variations between HD and MM.

**Additional file 4: Table S5.** The detailed information of MM patients and healthy donors (HD).

## Data Availability

All data generated or analyzed during this study, together with the Supplementary files, are included in this published article. Meanwhile, the datasets involved in the current study are available from the corresponding author on reasonable request.

## Abbreviations

MSCs: Mesenchymal stem/stromal cells
BM-MSCs: Bone marrow-derived MSCs
HD-MSCs: Healthy donor-derived MSCs
MM-MSCs: Multiple myeloma-derived MSCs
MDS: Myelodysplastic syndromes
AML: Acute myeloid leukemia
UC-MSCs: Umbilical cord-derived MSCs
ASCs: Adipose tissue-derived MSCs
ESC-MSCs: Embryonic stem cell-derived MSCs
iPSC-MSCs: Induced pluripotent stem cell-derived MSCs
DPSCs: Dental pulpal-derive stem cells
SCAP-Ss: Supernumerary teeth-derived apical papillary stem cells
KEGG: Kyoto encyclopedia of genes and genomes
PCA: Principal component analysis
COVID-19: Corona virus disease 2019
MM: Multiple myeloma
NDA: New drug application
HSCT: Hematopoietic stem cell transplantation
ITP: Immune thrombocytopenia
COPD: Chronic obstructive pulmonary disease
POF: Premature ovarian failure
ACLF: Acute-on-chronic liver failure
MLC: Mixed lymphocyte coculture
MACS: Magnetic activated cell sorting
DEGs: Differentially expressed genes
GOBP: Gene ontology biological process
GSEA: Gene set enrichment analysis
MMP-9: MM-derived matrix metalloproteinase-9
