# Supplementary material for: Characterization of the biological and transcriptomic landscapes of bone marrow-derived mesenchymal stem cells in patients with multiple myeloma
Source: Cancer Cell Int. 2024 Mar 27;24:116. doi: 10.1186/s12935-024-03308-2 (PMC10976750; doi:10.1186/s12935-024-03308-2)
Supplement: Supplementary file 1 — Additional file 1: Table S1. The list of the antibodies. Table S2. The primer sequences for q RP-PCR assay. [file 12935_2024_3308_MOESM1_ESM.docx]

**Supplementary Information**

**Characterization of the Biological and Transcriptomic Landscapes of Bone Marrow-derived Mesenchymal Stem Cells in Patients with Multiple Myeloma**

Yu Lu^1^**^§^**, Chaohui Zheng^2^**^§^**, Wenxia Zhang^1#^, Xuan Liu^1^, Ziwei Zhou^1^, Zhenzhen Wang^1^, Huan Hua^1^, Zhengrong Song^1^, Xuejun Zhang^1^, Shuyi Liu^1^, Leisheng Zhang^3,4*^, Fuxu Wang^1*^

**Supplementary Tables:**

**Supplementary Table S1. The list of the antibodies**

**Supplementary Table S2. The primer sequences for q RP-PCR assay**

**Supplementary Tables**

**Supplementary Table S1. The list of the antibodies**

**Table S3. Antibodies used in this study.**

Antibodies for flow cytometry.

| Antibody | Cat. NO. | Source |
| --- | --- | --- |
| Anti-CD3-PE-cy7 | 300419 | BioLegend |
| Anti-CD4-Percp | 550631 | BD Pharmigen |
| Anti-CD4-FITC | A07750 | BECKMAN COUNTER |
| Anti-CD183-APC | 561732 | BD Pharmigen |
| Anti-CD196-PE | 353409 | BioLegend |
| Anti-CD44-FITC | FITC-65063 | Proteintech |
| Anti-CD73-FITC | FITC-65162 | Proteintech |
| Anti-CD90-PE | B322608 | BioLegend |
| Anti-CD105-PE-cy7 | B43293 | BECKMAN COUNTER |
| Anti-IFN-γ-BV605 | 745111 | BD Pharmigen |
| Anti-HLA-DR-PE-cy7 | 335795 | BD Pharmigen |
| Anti-CD8-PE | A07757 | BECKMAN COUNTER |
| Anti-CD34-PE | PE-65183 | Proteintech |
| Anti-CD45-APC | APC-65109 | Proteintech |

**Supplementary Table S2 The primer sequences for qRP-PCR assay**

Real-time PCR primer sequences.

| Gene | Forward Primer | Reverse Primer |
| --- | --- | --- |
| *ACTIN* | CTCTTCCAGCCTTCCTTCCT | AGCACTGTGTGTTGGCGTACAG |
| *POU5F1* | CTTGAATCCCGAATGGAAAGGG | GTGTATATCCCAGGGTGATCCTC |
| *SOX2* | GCCGAGTGGAAACTTTTGTCG | GGCAGCGTGTACTTATCCTTCT |
| *NANOG* | TTTGTGGGCCTGAAGAAAACT | AGGGCTGTCCTGAATAAGCAG |
| *ADIPOQ* | TGGTCCTAAGGGAGACATCG | TGGAATTTACCAGTGGAGCC |
| *PPAR-γ* | GCTGGCCTCCTTGATGAATA | TGTCTTCAATGGGCTTCACA |
| *FABP4* | ACTGGGCCAGGAATTTGACG | CTCGTGGAAGTGACGCCTT |
| *RUNX2* | CTCACTACCACACCTACCTG | TCAATATGGTCGCCAAACAGATTC |
| *BGLAP* | GGCGCTACCTGTATCAATGG | TCAGCCAACTCGTCACAGTC |
| *COL1A1* | GAGGGCCAAGACGAAGACATC | CAGATCACGTCATCGCACAAC |
| *ACAN* | CCCCTGCTATTTCATCGACCC | GACACACGGCTCCACTTGAT |
| *SOX9* | AATGGAGCAGCGAAATCAAC | CAGAGAGATTTAGCACACTGATC |
| *COL2A1* | TGGACGATCAGGCGAAACC | GCTGCGGATGCTCTCAATCT |
